# Accuracy of Additively Manufactured and Milled Interim 3‐Unit Fixed Dental Prostheses

**DOI:** 10.1111/jopr.13454

**Published:** 2022-03-21

**Authors:** Duygu Karasan, Juan Legaz, Philippe Boitelle, Philippe Mojon, Vincent Fehmer, Irena Sailer

**Affiliations:** ^1^ Division of Fixed Prosthodontics and Biomaterials University Clinics for Dental Medicine, University of Geneva Geneva Switzerland; ^2^ Prosthodontic Department, Faculty of Dentistry Lille University, Lille University Medical Center Lille France; ^3^ Division of Removable Prosthodontics and Gerodontology University Clinics of Dental Medicine, University of Geneva Geneva Switzerland

**Keywords:** 3D printing, additive manufacturing, provisional restorations, interim restorations, fixed dental prosthesis

## Abstract

**Purpose:**

To investigate the accuracy of additive manufacturing (AM) by means of internal fit of fixed dental prostheses (FDPs) fabricated with two AM technologies using different resins and printing modes (validated vs nonvalidated) compared to milling and direct manual methods.

**Material and methods:**

Sixty 3‐unit interim FDPs replacing the first mandibular molar were divided into 6 groups (n = 10): manual (Protemp 4), milled (Telio‐CAD), and AM groups were subdivided based on AM technology (direct light processing (Rapidshape P30 [RS]) and stereolithography (FormLabs 2 [FL])) and the polymer type (P‐Pro‐C&B [St] and SHERAprint‐cb [Sh]) (RS‐St, RS‐Sh, FL‐St, FL‐Sh). Validated (RS‐Sh and RS‐St) or nonvalidated (FL‐St and FL‐Sh) modes were adopted for AM. The specimens were scanned to 3D align (GOM inspect) according to the triple scan method. The internal space between the FDPs and preparation surfaces in four sites (marginal, axial, occlusal, and total) was measured using equidistant surface points (GOM Inspect). Statistical analysis was done using Kruskal Wallis and Dunn post‐hoc tests. (α = 0.05).

**Results:**

One AM group (FL‐Sh) and milling exhibited better adaptation compared to manual and RS‐St at molar site (*p* < 0.05). FDPs with St resin (FL‐St and RS‐St) displayed bigger marginal space than milled, FL‐Sh, and RS‐Sh. The nonvalidated printing mode showed better mean space results (*p* < 0.05) with higher predictability and repeatability (*p* < 0.001).

**Conclusions:**

The AM interim FDPs tested provided valid alternatives to the milled ones in regard to their accuracy results. The printing mode, resin, and the AM technology used significantly influenced the manufacturing accuracy of interim FDPs, particularly at the marginal area. The nonvalidated printing mode with lower‐cost 3D printers is a promising solution for clinical applications.

Additive manufacturing (AM) is arguably one of the fastest developing technologies with great potential in the restorative/prosthodontic domain. With this expansion, a wider range of three‐dimensional (3D) printers and printable materials are being introduced to the market. This results in the creation of new clinical applications of the 3D printed products, thereby increasing demand and ultimately reducing costs of AM. The fabrication of interim restorations is one of the clinical applications that can benefit the most from the developments in 3D printing technology.

An interim restoration should offer certain mechanical stability, while maintaining the biological health of the teeth and their surrounding tissues.^1^, ^2^ Therefore, adequate 3D adaptation (up to a 125‐µm marginal and internal fit)^3^, ^4^ is essential but can be more difficult to achieve for multiple unit restorations. The milled computer aided design and computer aided manufacturing (CAD‐CAM) interim fixed dental prosthesis (FDPs) are validated clinical options as they offer adequate accuracy and mechanical stability.^5^ On the other hand, the usability of the AM technology for multiple unit FDPs remains questionable both in accuracy and mechanical stability aspects.

Even though 3D printing offers certain advantages over subtractive manufacturing, namely reduced initial cost of the device and waste material, the possibility of fabricating extended dental appliances such as dental models, as well as fabricating a large number of objects in a shorter time period,^6^ there are several parameters that can vary depending on the printer and/or to the material used.^7^, ^8^, ^9^, ^10^, ^11^, ^12^ A substantial amount of scientific literature has addressed the importance of factors such as laser speed, intensity, angle and build direction,^11^, ^13^, ^14^, ^15^ number of layers,^10^, ^16^ shrinkage between layers,^16^ amount of supportive material,^9^ and post‐processing procedures^16^ to achieve the most efficient and accurate modalities of AM. Having this myriad of parameters, the manufacturers of 3D printers often close their systems to be used only with validated printable materials in order to overcome potential inaccuracy and mechanical problems.

Vat‐polymerization is one of the most used AM methods for fabrication of dental devices, and can be subcategorized based on the light source employed on the printer: stereolithography (SLA), direct light processing (DLP), and liquid crystal display based (LCD) printers also called daylight polymer printing (DPP).^13^, ^17^, ^18^ Even though there are hypotheses regarding the possible differences between the resolution and accuracies of different vat‐polymerization methods, the closed systems offered by the 3D printer manufacturers make it difficult to compare the different printing technologies for specific indications.

Moreover, for the clinicians who prefer the use of AM as a chairside option instead of higher cost milling devices, there is unquestionably a demand for the use of low‐cost 3D printers that allow a wide range of material alternatives. Thus, investigations comparing so called “nonvalidated” manufacturing of the dental devices to the validated printing mode are needed.

Therefore, the purpose of this in vitro study was to evaluate the accuracy of AM by means of internal and marginal adaptation of interim FDPs that are fabricated using two different AM technologies employing validated and nonvalidated printing modes with different resins compared to the interim FDPs manufactured by subtractive CAM method and direct manual method. The null hypotheses were that the interim internal and marginal adaptation would not be influenced by the manufacturing method (manual, milling, and AM) and that the internal and marginal adaptation of the AM interim FDPs would not be influenced by the type of 3D printer, the printable resin used, or by the printing mode (validated and nonvalidated).

## Materials and methods

Three‐unit posterior interim FDPs were manufactured by means of manual, subtractive manufacturing (milling) and AM replacing the first mandibular molar. Two different vat‐polymerization 3D printers, DLP (P30; RapidShape, Straumann, Basel, Switzerland) and SLA (FormLabs 2; FormLabs, Somerville, MA), were used to print specimens out of two different commercially available printable resin materials. The six study groups (Mil, Man, FL‐Sh, FL‐St, RS‐Sh, RS‐St), materials used, and manufacturing specifications are detailed in Table 1.

**Table 1:** Material, manufacturing and post‐processing details of study groups

	Material Properties			Additive Manufacturing				Printing Parameters		Post Processing/Finalization		
Group	Name	Composition	Manufacturer	Technology	3D printer	Material/Nesting & slicing software	Printing mode	Layer thickness	Printing orientation	Cleaning	Post‐polymerization/Device	Final cleaning/Drying
Mil (n=10)	Telio CAD LT A2	Polymethylmethacrylate (PMMA) 99.5% and Pigments (<1.0%)	Ivoclar Vivadent, Schaan, Liechtenstein	Milling	5‐axis milling machine	Zenotec Select Hybrid, Wieland Dental, Forzheim, Germany	‐	‐	‐	‐	‐	‐
Man (n=10)	Protemp 4, A2	Dimethacrylate polymer. Bis‐GMA (Glycidyl methacrylate), zirconium particles, silica and silanes, pigments	3M ESPE AG, Seefeld, Germany	Direct molding	Polyvinylsioxane putty and light body	President Putty and Xtra Lightbody, Coltene Whaledent, Atstatten, Germany	‐	‐	‐	‐	‐	‐
FL‐Sh (n=10)	SHERA‐cb	Flowable, light‐curing acrylic‐based composite (Matrix: Methacrylate oligomers, Phosphine oxide. Proportions n.a)	SHERA Werkstoff‐ Technologie GmbH & Co, Lemförde, Germany	SLA	FormLabs 2, FormLabs, Somerville, MA, USA	PreForm Software, FormLabs, Somerville, MA, USA	Open Mode (non‐validated)	50µm	450 occlusal‐ buccal supported	2 x 4 mins ultrasonic bath (Unident Geneve, Geneva, Switzerland) with 98% Isopropyl alcohol (Sigma‐Aldrich, Merck KGaA, Darmstadt, Germany)	3 x 6000 flashes per occlusal and intaglio surfaces (SHERAFlash‐light plus, SHERA Werkstoff‐ Technologie GmbH & Co, Lemförde, Germany)	Compressed air and steam cleaning, support removal
FL‐St (n=10)	P Pro Crown & Bridge	Flowable, light‐curing acrylic‐based composite (Matrix: Methacrylate, Filler: Siliziumdioxid 50wt% and dental glass (30vol%))	Institut Straumann AG, Basel, Switzerland	SLA	FormLabs 2, FormLabs, FormLabs, Somerville, Somerville, MA, USA	PreForm Software, MA, USA	Open Mode (non‐validated)	100µm	450 occlusal‐buccal supported	Laboratory centrifuge, 1600 rpm for 2 x 2 mins	1 x 2000 flashes per occlusal and intaglio surfaces (SHERAFlash‐light plus, SHERA Werkstoff‐ Technologie GmbH & Co, Lemförde, Germany)	2 mins 98% Isopropyl alcohol (Sigma‐Aldrich, Merck KGaA, Darmstadt, Germany) brushing & compressed air cleaning & 30 mins drying, support removal
RS‐Sh (n=10)	SHERA‐cb	Flowable, light‐curing acrylic‐based composite (Matrix: Methacrylate oligomers, Phosphine oxide. Proportions n.a)	SHERA Werkstoff‐Technologie GmbH & Co, Lemförde, Germany	DLP	P30, RapidShape, Heimsheim, Germany	Autodesk Netfabb Standard 2020, Autodesk, Mill Valley, CA, USA	Validated	50µm	450 occlusal‐buccal supported	2 x 4 mins ultrasonic bath (Unident Geneve, Geneva, Switzerland) with 98% Isopropyl alcohol (Sigma‐Aldrich, Merck KGaA, Darmstadt, Germany)	3 x 6000 flashes per occlusal and intaglio surfaces (SHERAFlash‐light plus, SHERA Werkstoff‐ Technologie GmbH & Co, Lemförde, Germany)	Compressed air and steam cleaning, support removal
RS‐St (n=10)	P Pro Crown & Bridge	Flowable, light‐curing acrylic‐based composite (Matrix: Methacrylate, Filler: Siliziumdioxid 50wt% and dental glass (30vol%))	Institut Straumann AG, Basel, Switzerland	DLP	P20II, RapidShape, Heimsheim, Germany	Autodesk Netfabb Standard 2020, Autodesk, Mill Valley, CA, USA	Validated	100µm	450 occlusal‐buccal supported	Laboratory centrifuge, 1600 rpm for 2 x 2 mins	1 x 2000 flashes per occlusal and intaglio surfaces (SHERAFlash‐light plus, SHERA Werkstoff‐ Technologie GmbH & Co, Lemförde, Germany)	2 mins 98% Isopropyl alcohol (Sigma‐Aldrich, Merck KGaA, Darmstadt, Germany) brushing & compressed air cleaning & 30 mins drying, support removal

DLP, direct light processing; SLA, stereolithography; n.a, not available; rpm, revolutions per minute; min, minute

A mandibular typodont model (AG‐3; Frasaco GmbH, Tettnang, Germany) was modified by removing the tooth #46 to create an edentulous area. Teeth #45 and #47 then were prepared following the preparation principles for complete‐coverage restorations.^19^ Circumferential 1mm chamfer finish line design, 1.5 mm occlusal and 1mm axial reductions were employed for both premolar and molar teeth. A digital impression was obtained from the typodont model by using an optical laboratory scanner (Iscan D104i; Imetric 3D SA, Courgenay, Switzerland). The digital files were exported in standard tessellation language (STL) format and were then processed in a model processing software program (Dental System, Model Builder; 3 Shape, Copenhagen, Denmark). Later the model was 3D printed (P30; Rapidshape, Straumann, and SheraPrint Model Plus Sand; SHERA Werkstoff‐Technologie GmbH & Co) to be used as the master model. The rationale behind obtaining the master model from a 3D printed material was to ensure the similar surface characteristics and light reflection properties between the specimens and the master model.

A digital impression of the AM master model was taken (Iscan D104i, Imetric 3D SA, Courgenay, Switzerland) and FDPs were CAD (Dental System, version 2018; 3 Shape) in a full contour shape with 1mm of marginal and axial wall thickness and, 1.5 mm of occlusal thickness. The overall cement space was set to 30 µm^20^ with an additional vertical space of 80 µm. Both the CAD data and the CAD‐master model assembly were exported. The latter was 3D printed (P30; RapidShape and SheraPrint Model Plus Sand; SHERA Werkstoff‐Technologie GmbH & Co) and the silicone indexes (President Putty and Xtra Lightbody; Coltene Whaledent, Altstatten, Germany) were created on it for the manually manufactured interims (Man group). Manual specimens were manufactured with the direct molding method (Table 1). Self‐polymerizing interim material was applied in a silicon index and successively pressed on the master model. The silicon index was removed after 6 min of working/setting time following manufacturer's recommendations; the FDPs were then removed and the excessive resin material around the margin was detached carefully. The intaglio surface as well as the marginal area remained untouched.

The milled and four AM group specimens were fabricated using the same STL file. The milled FDPs (Mil) were nested and milled with a CAM unit (Table 1). Specimens of four AM groups (RS‐Sh, RS‐St, FL‐Sh, FL‐St) were fabricated and post‐processed. For the RS‐Sh and RS‐St group specimens a validated 3D printing mode was used, whereas the FL‐Sh and FL‐St specimens were manufactured using open mode option of the FormLabs 2 3D printer; the printer was set to “white” as the material color and the layer thicknesses were set based on manufacturer's recommendations. The pre‐processing, processing and post‐processing methods applied to the AM group specimens are detailed in Table 1.

**Figure 1:**
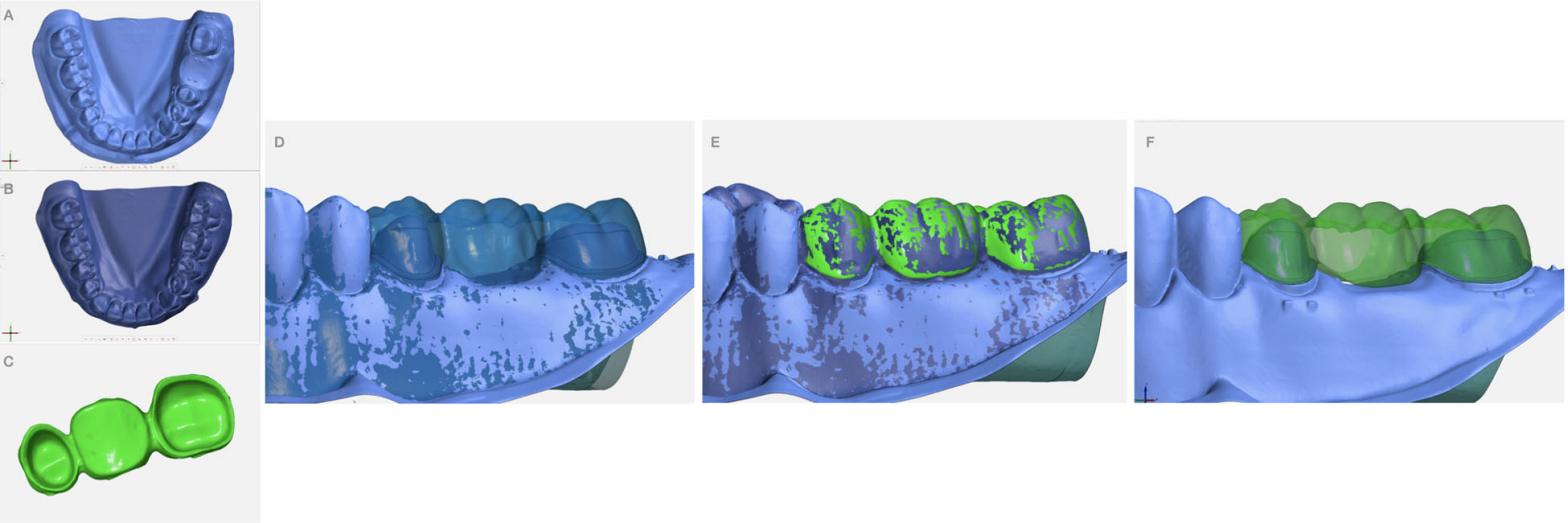
The scan data and superimposition of the scans according to triple scan method based on “Best‐fit Alignment” in a software program (GOM Inspect, GOM GmbH). A, Master model (preparation scan). B, FDP positioned on the master model (assembly scan). C, Intaglio and outer surface of the FDPs (FDP scan). D, Superimposition of the preparation and assembly scans. E, Superimposition of the FDP scan to the assembly scans. F, FDP and preparation scans aligned. FDP = fixed dental prosthesis.

The master model and the 3‐unit FDPs were scanned using an optical laboratory scanner (Iscan D104i; Imetric 3D SA). An anti‐reflective powder coating (Helling 3D Scan Spray; Helling GmbH) was sprayed at a fixed distance of 20 cm on the preparation, intaglio and outer surfaces of the specimens. The accuracy of the scanner was reported as 4 µm by the manufacturer. The scanning was made following a modified triple scan method^21^: the scanning of the preparation (preparation scan); scanning of the specimen, intaglio and outer surface of the FDPs (FDP scan); scanning of the assembly, and the FDP was positioned on the master model (assembly scan) (Fig 1). The scan data were exported as STL files to be then imported in a reverse engineering software program (GOM Inspect, V8 Hotfix 5, Rev. 115656, Build 2019‐02‐15, GOM GmbH, Carl. AG, Brunswick, Germany) for the analysis.

**Figure 2:**
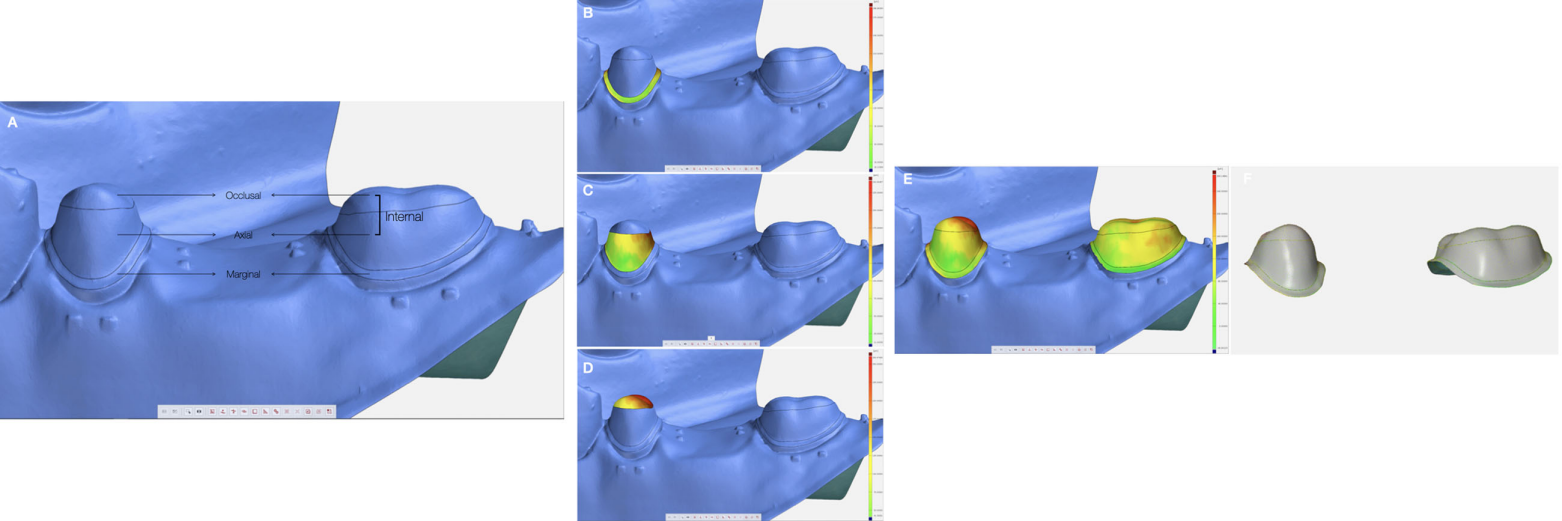
A, Inspection sites that were defined as three areas: marginal, axial, and occlusal. B‐D, Measurement of the spaces between the intaglio surface of the FDP scan and preparation scan surface (B, marginal site; C, axial site; D, occlusal site). E, 3D analysis of the total surface. F, The equidistant surface points that were used for the space measurements.

Initially, the preparation scan was imported, and the abutment surfaces of the preparation scan were separated applying three surface curves and corresponding sections in the inspection software (GOM Inspect; GOM GmbH) to create 3 areas‐‐ marginal, axial and occlusal (Fig 2). The marginal area was defined by two curves; the first was positioned at the cavosurface angle and the second at the intersection between the finish line and axial wall of the preparation. A curve separating axial and occlusal areas was positioned where functional and nonfunctional cusp bevels end at the buccal and lingual aspects, and in the approximal areas at the intersection between occlusal and axial preparation walls. Surface patches were created for the three inspection sites (marginal, axial, and occlusal) on the preparation scan. The inspection surface area thus could be standardized for each specimen for every site and tooth (Fig 2). Following the sectioning preparation scan, the assembly scan was imported and pre‐aligned to the preparation scan. Based on this pre‐alignment, the surfaces excluding the experimental area (preparation and the FDP surfaces) were selected on the actual data and section‐based “Best Fit Alignment” was performed. The FDP scan was then aligned to the assembly scan by using the same alignment strategy. The occlusal, buccal, and lingual outer surfaces of the scan were selected for the “Best Fit Alignment”. The alignment deviation was calculated within the software as <5 µm for both “Best Fit Alignments” (preparation scan to assembly scan and assembly scan to FDP scan) during each analysis (Fig 1).

The internal and marginal adaptation of the FDPs at three defined areas (marginal, axial, and occlusal) for each abutment tooth (premolar and molar) were measured at multiple points throughout the surface using the surface comparison option (GOM Inspect; GOM GmbH) (Fig 2). The number of measurement points were 1208 and 1953 at the occlusal area, 1404 and 1913 at the axial area, and 1023 and 1649 at the marginal area on the premolar and molar teeth, respectively (Fig 2). The inspection was limited to 500 µm and the exceeding measurements were considered as outliers.

The single point measurements were exported to a software program (Microsoft Excel; Microsoft, Redmond, Washington, United States) and were used to calculate the mean space between the marginal, intaglio (axial and occlusal), and total areas of the abutment teeth. The standard deviations for each specimen per inspection site were also calculated (SD_Spe_) which corresponded to the level of homogeneity of the internal and marginal space throughout the inspection surfaces.

Selected specimens from AM groups were analysed for layer, bulk, and surface characteristics using a scanning electron microscope (SEM) (X11, X25, X50, and X200, Gemini Zeiss Sigma 300 VP; Carl Zeiss, Oberkochen, Germany) (Figs 4 and 5). Real layer thickness to evaluate the z‐axis accuracy was measured in the selected specimens when the layers were visible under magnification (Fig 4).

The mean, standard deviation (SD), median and interquartile range (IQR) of mean internal (occlusal and axial) and marginal spaces and their corresponding mean deviations for study groups were analyzed using descriptive statistics. The mean internal and marginal spaces were tested for normality by Shapiro‐Wilk test. Kruskal‐Wallis test was used for comparisons of internal and marginal mean spaces in between the groups. A Dunn post hoc test was later applied to detect the differences across the groups for each site of abutment teeth (*p* < 0.05).

A general linear model using mean deviations as the dependent variable and tooth type and tooth site as fixed effect factors was run. One‐way analysis of variance (ANOVA) test was further performed to highlight differences between the groups for total surface mean deviations. Tukey B post‐hoc test was done to identify the groups that shows significant differences. (*p* < 0.05). Mann Whitney U and t‐tests were used to analyze and compare the total 3D adaptation and the mean deviations of the AM interim FDPs fabricated by two 3D printers. The statistical analysis was performed using a statistical software program (IBM SPSS Statistics, v24.0; IBM Corp, New York, NY)

## Results

Descriptive statistics for 3D adaptation results of specimens from 6 study groups, four inspection sites (marginal, axial, occlusal, and total), and two abutment teeth (premolar and molar) are shown in Table 2.

**Table 2:** Group, tooth, and site descriptive statistics for mean distance [µm], SD, median, IQR and Dunn* test results

																	Total				
Site / Tooth	Group n=10	Mean distance	SD	Marginal median	IQR	Dunn test comparison	Mean distance	SD	Axial median	IQR	Dunn test comparison	Mean distance	SD	Occlusal median	IQR	Dunn test comparison	Mean distance	SD	Median	IQR	Dunn test comparison
Premolar	Mill	117.60	62.65	118.74	58.11	a	109.72	39.77	104.33	25.26	a	153.74	70.41	137.15	62.64	a, b	143.55	48.68	127.97	47.11	a
	Man	165.15	38.89	169.11	60.27	b, c	90.49	26.15	92.69	30.76	a	142.38	44.28	138.65	60.64	a, b	128.04	31.04	132.41	38.24	a
	FL‐Sh	103.07	18.01	102.17	29.38	a	101.18	6.81	100.38	6.86	a	169.48	52.14	165.17	30.26	b	117.07	13.71	118.78	24.54	a
	FL‐St	172.50	30.70	180.17	42.48	c	108.08	15.07	109.99	20.61	a	116.29	28.23	109.57	33.73	a	130.83	22.81	133.28	33.35	a
	RS‐Sh	130.92	27.58	129.04	28.21	a, b	100.13	16.40	101.95	15.36	a	155.33	23.17	157.06	42.25	b	125.25	19.83	126.77	16.18	a
	RS‐St	160.27	33.69	158.19	34.66	b, c	122.60	25.14	115.46	29.27	a	168.23	36.33	159.83	37.42	b	147.82	29.96	142.09	27.59	a
Molar	Mill	83.31	68.72	49.51	28.77	a, b	93.94	50.45	73.68	17.3	a, b	113.11	82.95	79.14	41.49	a, b	98.85	63.95	74.42	28.73	a
	Man	188.38	73.76	178.91	90.13	d	101.7	46.89	89.79	69.03	a, b, c	150.78	73.74	138.72	98.95	b, c	170.98	105.47	141.99	119.37	b
	FL‐Sh	69.10	23.25	68.09	24.85	a	67.92	14	68.04	14.87	a	103.03	23.79	103.73	34.21	a	79.48	18.2	79.62	14.17	a
	FL‐St	114.76	17.13	109.97	31.04	c, d	98.28	13.13	100.31	13.79	c	67.6	8.84	67.41	9.96	a	97.27	11.5	97.87	13.78	a, b
	RS‐Sh	102.35	31.84	96.29	23.74	b, c	79	20.5	76.88	15.27	a, b	128.12	37.17	120.13	29.21	b, c	101.91	28.22	94.28	14.78	a, b
	RS‐St	110.52	24.30	111.96	30.06	c, d	85.54	11.85	82.45	9.49	b, c	131.27	11.74	132.43	5.27	c	107.98	15.53	106.3	14.63	b

*Dunn test comparison was done intergroup for each tooth and measurement site

Note: the same letter denotes no significant difference (p>.05)

SD, standard deviation; IQR, interquartile range; Mil, milled; Man, manual; FL‐Sh, FormLabs 2 and Shera‐cb; FL‐St, FormLabs 2 and P‐Pro Crown & Bridge; RS‐Sh, P30 Rapidshape and Shera‐cb; RS‐St, P30 Rapidshape and P‐Pro Crown & Bridge

Marginal space results of FL‐Sh and RS‐Sh groups were similar to Mil group and exhibited significantly better marginal adaptation than FL‐St, RS‐St, and Man groups for both premolar and molar abutment teeth analysis (Table 2). The resin type showed a significant influence on the marginal adaptation, for the SLA printer specimens (FL‐Sh < FL‐St, *p* < 0.05).

**Figure 3:**
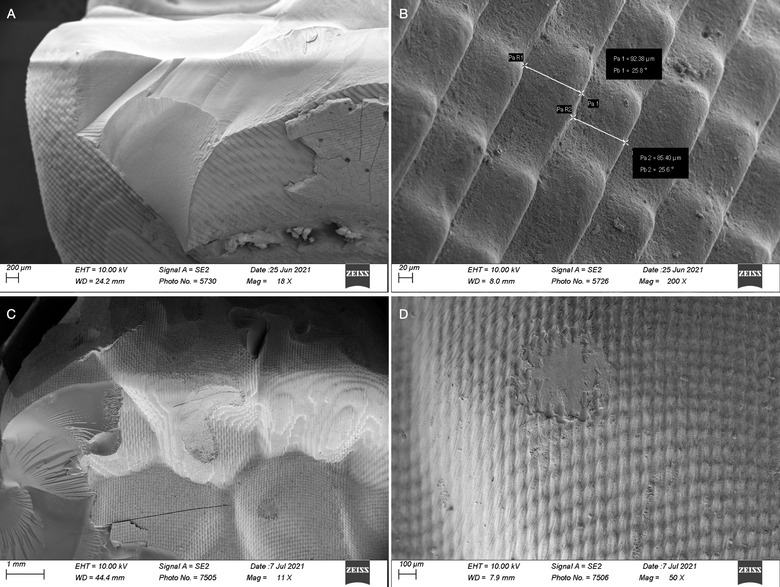
SEM images of RS‐St (A, B) and RS‐Sh (C, D) (×11, ×18, ×50, ×200 magnifications); A, The pixel like appearance of the layers at the surface. B, The measurement of layer thickness resulted in variant values from the original setting of 100 µm. C, The occlusal image of a selected RS‐Sh specimen, the pixel like appearance of the surface. D, The occlusal surface of the same RS‐Sh specimen with a higher magnification (×200). SEM = scanning electron microscope; RS‐Sh = P30 Rapidshape and Shera‐cb.

Fabrication method (milling, manual, or AM), and the interim material influenced the total 3D adaptation of the interim FDPs at the molar abutment. Mil and FL‐Sh specimens were significantly better adapted than Man and RS‐St specimens (*p* < 0.05) (Fig 3). The total surface adaptation results of 3D printers (FL and RS) were pooled in order to compare the accuracy of the two 3D printers (Table 3). The FL interim FDPs showed a trend for better overall 3D adaptation results compared to RS specimens, however the difference reached significance only at the molar site based on Mann‐Whitney U test results (U (20,20) = 281, *p* < 0.05) (Table 3).

**Table 3:** Mean and standard deviation results and comparison of total surface and SDspe analysis of two AM devices (FormLabs 2 and P30 Rapidshape)

Total surface [mean]							
Device	Tooth	Specimen [n]	Mean [µm]	SD	Median [µm]	IQR	Significance
FL	Premolar	20	123.9	19.6	124.1	30.5	t(38) = 1.67 /
RS	Premolar	20	136.5	27.3	130.6	30.9	p > .05 *
FL	Molar	20	88.4	17.4	89.8	27.0	U(20,20) = 281 /
RS	Molar	20	104.9	22.4	99.2	23.7	**P < .05** **

Total surface [SD*_S_* *_pe_*]							
Device	Tooth	Specimen [n]	Mean [µm]	SD	Median [µm]	IQR	Significance
FL	Premolar	20	65.5	8.9	65.7	14.2	t(38) = −3.52 /
RS	Premolar	20	76.0	9.7	74.2	11.7	**P < .01** *
FL	Molar	20	60.2	9.2	60.2	9.1	U(20,20) = 114 /
RS	Molar	20	55.6	9.5	53.6	8.9	**P < .05** **

* t‐test results.

** Mann‐Whitney U test results.

SD = standard deviation; IQR = interquartile range; AM = additive manufacturing. FL = FormLabs 2; RS = P30 Rapidshape

The mean and SD of the SD_*Spe*_ for the total 3D adaptation analysis is shown in Table 4. Mil specimens showed significantly less total surface *SD_Spe_*, and therefore more homogenous internal and marginal space distribution compared to Man and RS‐St interim FDPs. Meanwhile despite their higher SD_*Spe*_ results, FL‐Sh, FL‐St, and RS‐St interim exhibited statistically similar results to Mil interim FDPs (Fig 3). The analysis on the pooled SD_*Spe*_ based on AM device, demonstrated significantly more homogenous total internal and marginal space distributions for FL 3D printer at both premolar and molar sites, according to t‐test and Mann‐Whitney U test results, respectively (Table 3).

**Table 4:** Descriptive statistics for SD_*S* *pe*_ of the total abutment teeth surface (premolar and molar pooled)

	Total		
Group n = 10	SD_*S*__*pe*_	SD	Tukey B comparison
**Mill**	52.9	24.8	**a**
**Man**	73.7	24.8	**b, c**
**FL‐Sh**	60.9	10.2	a, b, c
**FL‐St**	64.8	8.3	a, b, c
**RS‐Sh**	63.3	12.9	a, b, c
**RS‐St**	68.2	14.9	**b, c**

Tukey test comparison was done intergroup for FDP internal and marginal area; the same letter defines no significant difference (*p* > 0.05).

SD = standard deviation; SD_*S* *pe*_ = standard deviations for each specimen. Mil = milled; Man = manual; FL‐Sh = FormLabs 2 and Shera‐cb; FL‐St = FormLabs 2 and P‐Pro Crown & Bridge; RS‐Sh = P30 Rapidshape and Shera‐cb; RS‐St = P30 Rapidshape and P‐Pro Crown & Bridge.

**Figure 4:**
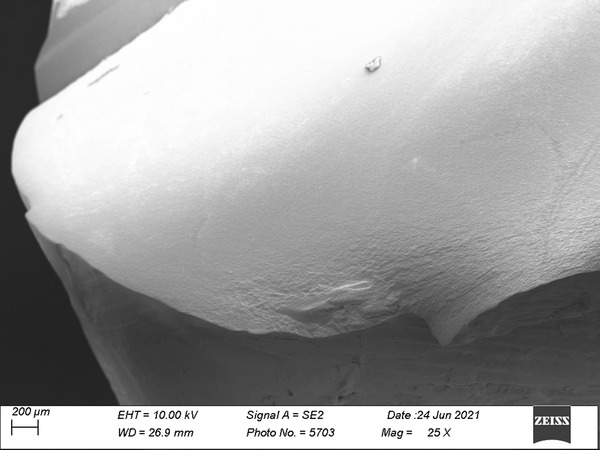
The surface image of a selected FL‐St specimen under SEM (×25). SEM, scanning electron microscope. FL‐St = FormLabs 2 and P‐Pro Crown & Bridge.

**Figure 5:**
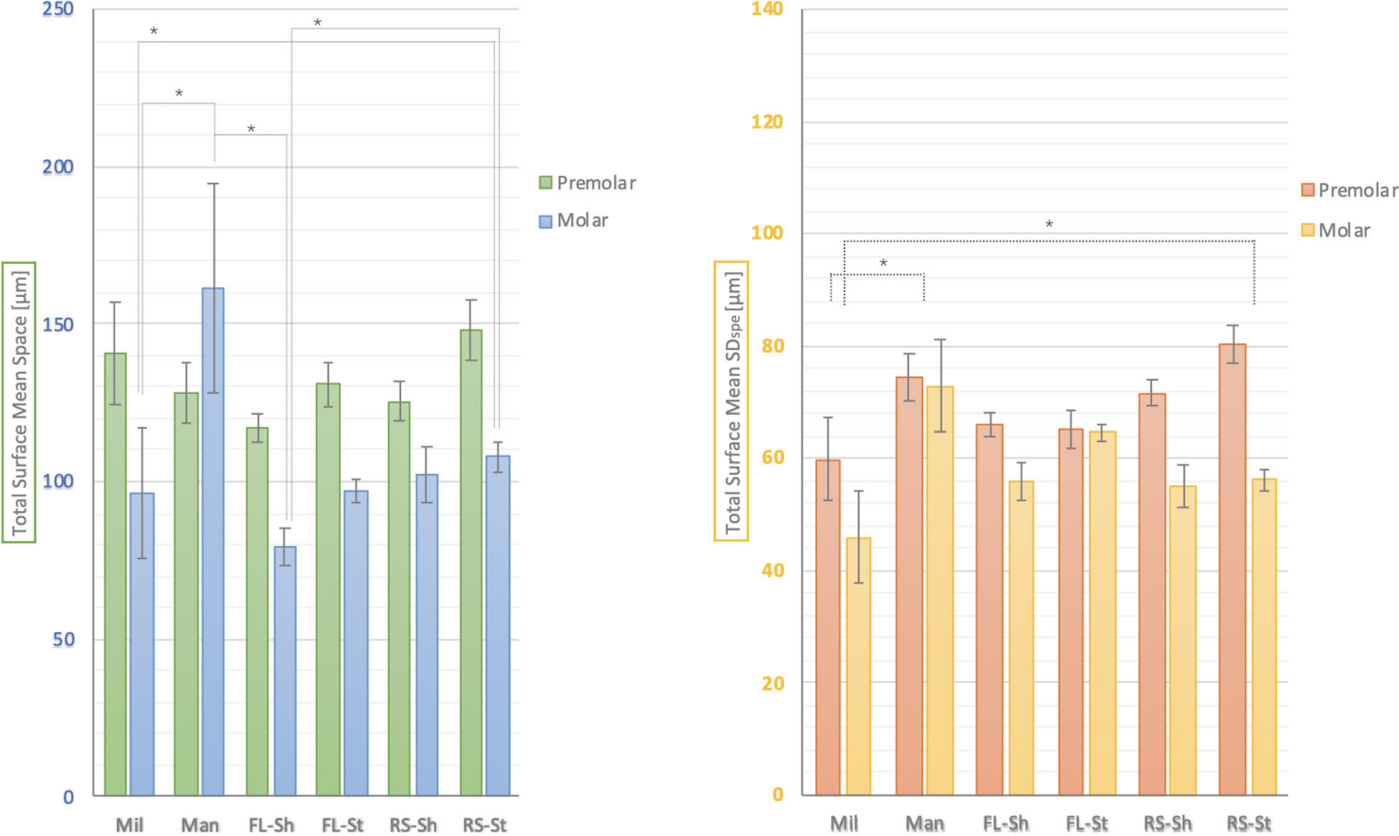
Total surface mean space and SD_*S* *pe*_ results for premolar and molar tooth areas (µm). SD_*S* *pe*_, standard deviations for each specimen.

Based on the SEM analysis on the selected specimens, the SLA printer specimens demonstrated smoother surface characteristics (Fig 5) compared to specimens fabricated with the DLP 3D printer (Fig 4). During the SEM analysis, the DLP printer specimens’ printing layers were detectable (Fig 4), whereas the SLA printer specimens’ layers were not visible.

## Discussion

Based on the findings of the present study the total adaptation results at the molar site of milled and one AM group (FL‐Sh) exhibited better adaptation results compared to Man and RS‐St groups. The difference between the manufacturing methods at the premolar site was not significant. Therefore, the first null hypothesis was partially rejected. Similar if not smaller marginal mean space results were obtained from the FL‐Sh group compared to Mil specimens, thus AM can be regarded as a promising alternative to milling for interim FDPs.

Previous investigations on comparison of the accuracy of milling and AM reported similar if not better marginal and internal adaptation for AM interim restorations,^23^, ^24^ which is in accordance with the findings of the present study. However, in the present study, not all the AM groups showed similar accuracy to the Mil group, and the accuracy of the AM groups were dependent on the AM technology, resin that was used, and their corresponding printing parameters. Therefore, the second null hypothesis was rejected. The AM groups matched with St resin material displayed less favorable marginal adaptation results compared to the Mil, FL‐Sh, and RS‐Sh groups. The reason behind this difference might be the layer thickness that was employed based on each manufacturer's recommendations (Sh: 50 µm, St: 100 µm).

The printer accuracy is determined by the resolution of the x‐, y‐, and z‐axis, which is related to the characteristics of the printer's light source. Contrary to the x‐ and y‐axis resolution, the resolution along the z‐axis is modifiable depending on the material and determines the layer thickness. The z‐axis resolution is reported to influence the degree of conversion which showed better results with lower layer thicknesses and eventually reduced the distortion due to photopolymerization shrinkage.^18^ In the present study, the resin with bigger layer thickness exhibited less favorable accuracy results independent from the AM technology used. Moreover, the layer thickness measurement under SEM for the DLP printer specimens, demonstrated variant values compared to the layer thickness setting that was chosen as can be seen in the SEM images.

Printing parameters such as layer thickness, shrinkage between layers,^7^, ^25^ layer number,^12^, ^25^ laser intensity, printer wavelength, post‐processing method, and UV intensity and total thickness^26^ were suggested to have influence on the distortion phenomenon of the printed object. Customizing printing parameters according to the resin used was suggested as a solution.^26^ However, there is an obstacle in dentistry, as existing 3D printer manufactures (i.e., NextDent, Rapidshape and DWS) often market devices that are only compatible with their respective printable dental materials. These are often expensive >$50,000 printers with high production capacity.^12^ Nevertheless, there is a widespread need for lower‐cost (<$5000) 3D printers as used in this study (e.g., FormLabs 2) as an alternative to the more expensive ones (e.g., Rapidshape P30) for easier access to 3D printing. This creates the need for investigations testing the accuracy of printing when used with nonvalidated printable resins. In the present study the SLA 3D printer group specimens (FL‐Sh and FL‐St) were manufactured using the nonvalidated printing mode, which means that the printer was employed in open‐mode. Whereas the DLP 3D printer (RS‐Sh and RS‐St) was used only with validated printable resins. Interestingly, the nonvalidated printing mode outperformed the validated counter group in premolar and molar tooth areas when both mean spaces and the SD_*S* *pe*_ were compared. In other words, the nonvalidated printing mode showed higher mean accuracy results with better predictability and repeatability. The mechanical stability results based on the second part of this investigation^22^ were in accordance with the results of the present study; the SLA technology with nonvalidated printing mode exhibited higher survival rate and lower complication rates after aging, as well as higher fracture load values compared to the specimens fabricated with the DLP printer with validated printing mode.^22^

Additionally, the influence of the AM technology (i.e., SLA or DLP), on the accuracy of AM dental devices should not be overlooked.^7^, ^8^, ^23^, ^25^ DLP 3D printers employ a digital light projector which might have different levels of resolution that directly define the accuracy of the printer. The pixels that are generated by the projector can be seen clearly in the SEM analysis that was performed in the present study. SLA printers have a laser beam that moves throughout each layer and printer accuracy depends on the laser spot diameter. The continuous photo‐polymerization of the resin makes the layers untraceable under the SEM for FL‐St and FL‐Sh and results in better surface finish than DLP.^6^ Similarly, the SLA demonstrated better accuracy at the marginal area and predictability of results than the DLP.

The triple scan method is a validated nondestructive alternative to the replica method.^21^, ^27^, ^28^ In the present study, the superimpositions and the analysis wwere done based on a modified triple scan method that allowed measurements on more than 6000 surface points that were distributed to the inspection area. For the replica method, the optimal number of points for the marginal fit was recommended to range from 18 to 50, however most authors reportedly used only 4 to 12 points. The triple scan method on the other hand may be done based on a higher number of points (range of 200‐300)^29^ yet remains limited compared to the present analysis protocol with 3D analysis of the space. Accordingly, this method provides a global cement space analysis and therefore the potential for an in‐depth analysis of the accuracy performance of CAD‐CAM systems and manufacturing workflows.

The results of this study should be interpreted with care as the in vitro design has limitations to reflect all clinically relevant factors, the limited number of interim dental materials and manufacturing procedures tested, and the specific setting printing parameters. Further in vitro and clinical trials are needed to broaden the analysis of the usability of additively manufactured interim FDPs.

## Conclusions

Within the limitations of this in vitro study, the AM interim FDPs demonstrated acceptable accuracy results, similar to milled ones. The printing mode, resin, and the printing technology used have influenced the printing accuracy of the interim FDPs, particularly at the marginal area. The use of nonvalidated printing mode with lower‐cost 3D printers, can be an interesting solution for clinical applications.

## Conflict of interest

Authors declare no conflict of interest.
